# Joubert syndrome a rare entity and role of radiology: A case report

**DOI:** 10.1016/j.amsu.2022.104113

**Published:** 2022-06-30

**Authors:** Irfan Ullah, Kiran Shafiq Khan, Rifayat Ullah Afridi, Farida Shirazi, Irum Naz, Aneela Ambreen, Manjeet Singh, Muhammad Sohaib Asghar

**Affiliations:** a Department of Pediatrics, Naseer Teaching Hospital, Peshawar, Pakistan; b Kabir Medical College, Gandhara University, Peshawar, Pakistan; c Liaquat National Hospital and Medical College, Karachi, Pakistan; d Dow University of Health Sciences, Karachi, Pakistan

**Keywords:** Joubert syndrome, Hypotonia, Cerebellum abnormality, Case report

## Abstract

**Introduction and importance:**

Joubert syndrome (JS) is defined by the characteristic set of cerebellum and midbrain abnormalities that communally result in the indicative “molar tooth sign” on the axial MRI report. The incidence of estimated to be from 1:80,000 to 1:100,000.

**Case presentation:**

Clinical features can be noticed shortly after birth that includes hypotonia episodic tachypnea and apnea that may be followed by developmental delays and speech apraxia. Polydactyly, cleft lip or palate, tongue abnormalities, hypotonia, encephalocele, meningocele, hydrocephalus, kidney problems, pituitary abnormality, and autistic-like behavior are the other deformities that can be seen with JS. Seizures may also occur. Motor disability and mental health range from mild to severe forms.

**Clinical discussion:**

Treatment for JS is symptomatic and supportive. The prognosis depends on cerebellar vermis development.

**Conclusion:**

JS can be missed if special attention were not given to radiological findings.

## Highlights

• Joubert syndrome (JS) is rare entity characterized by set of cerebellum and midbrain abnormalities.
• This case study highlighted a 17-month-old baby presented with typical JS features diagnosed radiologically.
• The patient was given multivitamins, and the parents were counseled, educated on other supportive therapies.

## 1 Introduction

Joubert Syndrome (JS) is a rare autosomal recessive syndrome, the first case of Joubert syndrome was first identified by Marie Joubert in 1969 [1]. JS is characterized by hypotonia, ataxia, oculomotor apraxia, facial dysmorphism, irregular neonatal breathing, and specific mid-hindbrain malformation “molar tooth sign” [[2], [3], [4]]. Molar tooth sign is a radiological hallmark that shows cerebellar vermis hypoplasia or dysplasia, thick and horizontally oriented superior cerebellar peduncles, and abnormally deep interpeduncular fossa [[5], [6], [7]]. Occipital encephalocele, polymicrogyria, polydactyly, ocular coloboma, retinal dystrophy, cystic kidney disease, nephronophthisis, and hepatic fibrosis all are termed as JS related disorders [8]. The reported incidence of JS is between 1:80,000 and 1:100,000 [9,10]. Joubert syndrome can be sporadic or hereditary [10]. In this case, we report a 17-month-old baby presents with typical JS features.

## 2 Case presentation

**Fig. 1:**
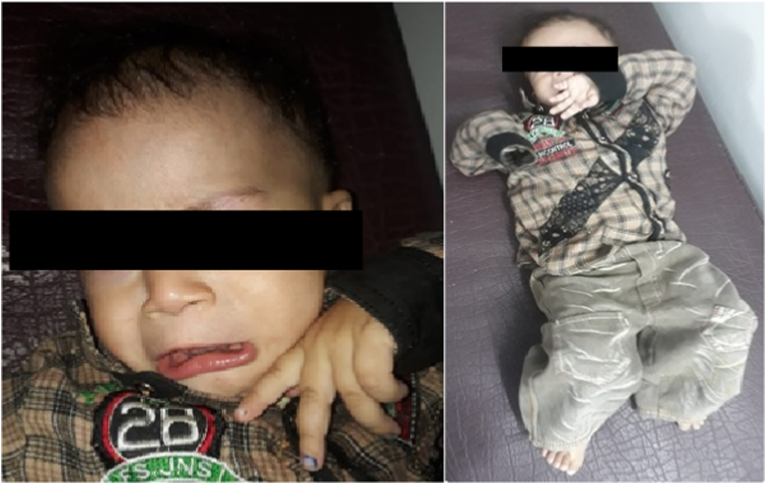
Physical appearance of the patient.

A 17-month male baby born to nonconsanguineous parents presented with delayed milestones (motor and speech delay) (Fig. 1). Birth history was uneventful (Full term, no perinatal event) and C-section, at hospital. Two siblings (sisters) were normal. The head circumference and weight were 46 cm and 8 kg respectively. On physical examination the patient was floppy, with decreased reflexes, decreased power and decreased tone (hypotonia), tongue fasciculations, and downgoing planters.

**Fig. 2:**
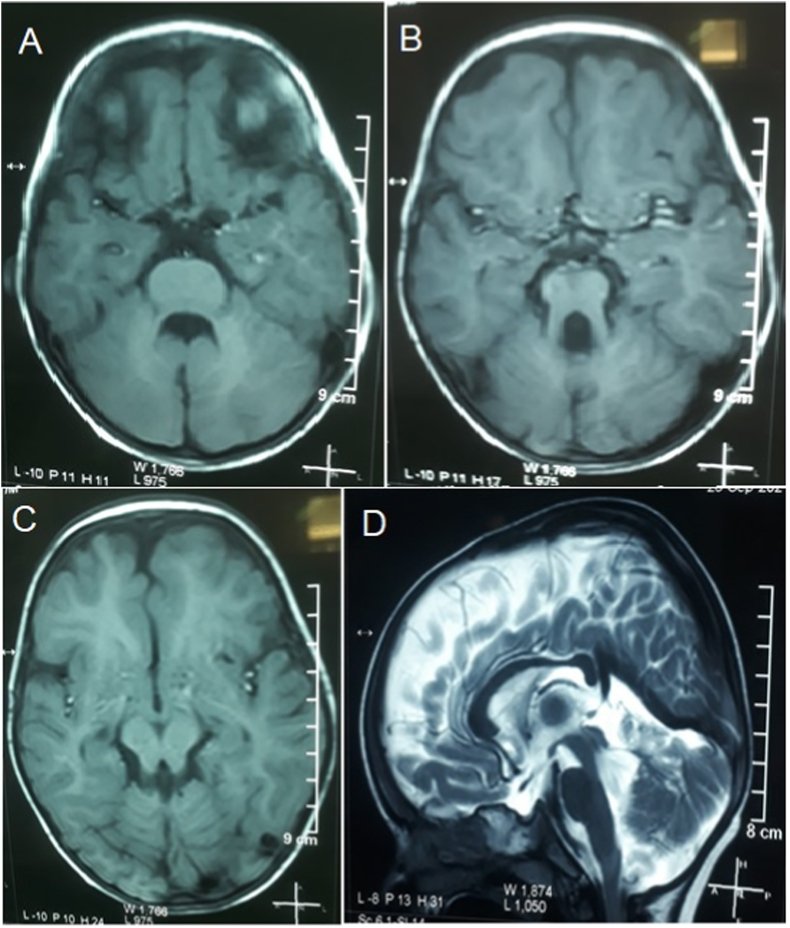
Radiological findings characteristics of Joubert syndrome.

Laboratory report shows Hb 9.7 g/L, TLC 7.4 x10^9^/L, Platelets 203 x10^9^/L, TSH 3.37 μIU/mL, T3 2.10 pg/ml, T4 125.11 nmol/L, and CPK 33 U/L. He was previously considered a case of cerebral palsy. Abdominal ultrasound was normal. Nerve conduction studies and Electromyography shows normal findings (no evidence of any peripheral neuropathy, spinal muscle atrophy or Myopathy). Magnetic resonance imaging (MRI) of Brain showed Hypoplasia of cerebellar vermis and superior cerebellar peduncle with fourth ventricle giving bat wing sign (Fig. 2A). Prominent thickened elongated cerebellar peduncle giving molar tooth appearance (Fig. 2B). Prepontine mesencephaly quadrigeminal system is enlarged suggesting midbrain atrophy (Fig. 2C). CSF space around vermis enlarged suggesting vermis atropy (Fig. 2D). Based on clinical findings and MRI, patient was diagnosed as case of Joubert Syndrome and put on multivitamins. Parents were counseled in detail. Informed consent was taken from the patient's guardian for the case report. This case report has been reported in line with the SCARE Criteria [11].

## 3 Discussion

Clinically Joubert syndrome (JS) is characterized by three primary findings; a typical cerebellar and brain stem malformation i.e. molar tooth sign (MTS), hypotonia, and developmental delays [12]. These findings are often followed by episodic tachypnea or apnea and peculiar eye movements. Breathing abnormalities may improve with time, truncal ataxia develops over time. A gross motor milestone delay has been observed in a patient with JS [10]. In several studies, hypotonia is regarded as one of the major signs [[12], [13], [14]]. Maria et al. confirmed it in a study conducted among 59 patients and all included cases presented with hypotonia [15]. Similarly, our patient has decreased reflexes, decreased power, decreased tone (hypotonia), tongue fasciculations, and downgoing planters. Other differentials such as Dekaban-Arima syndrome, Senior-Loken syndrome, COACH syndrome, and Varadi-Papp syndrome could be considered [16]. Radiological diagnosis of JS may include MRI scan, retinal examination, renal ultrasonogram, electroretinogram, and Karyotyping, but most of the cases were diagnosed on radiographic findings [16].

The patient with JS has hyperventilating breathing pattern, more in the awake state when the patient is stimulated, interspersed with central apnea. This abnormal breathing pattern is found more typically in the neonatal period and wanes by 11 years of age. In a study conducted by Maria et al. 71% of individuals have abnormal breathing patterns [15]. Pellegrino et al. stated 68% and in a study conducted by Kendall et al. 44% reported abnormal breathing patterns in patients with JS [16,17]. Our patient didn't develop any respiratory complications.

Abnormal ocular movement particularly nystagmus and ocular apraxia has been reported in a patient with JS [16]. Nystagmus and ocular apraxia may present at birth and improve with age [16]. Other visual findings include strabismus, ocular coloboma, severe visual loss, ptosis, and pigmentary changes in the fundus [18,19]. On contrary, our patient didn't have nystagmus at birth nor developed it later.

Our patient didn't have any cerebral pathology. Although vermian cleft, MTS, and “bat wing” signs were noticed radiologically. Multi-organ involvement has been reported in JS such as retina, liver, and kidney. This involvement of organs is termed ciliopathic syndromes. No curative therapy is available to date [16]. Therefore, early diagnosis and close follow-up are important for a beneficial outcome.

To date, 34 pathogenic variants have been reported, 33 of them are autosomal recessive and 1 is X-linked [10]. Around 62–94% of patient diagnosis is supported by the presence of biallelic pathogenic variants [20]. A number of genes have been identified as contributing to JS. Mutation in 13 ciliary or basal body gene account for 50% of the JS related disorders (JSRD) [21]. In a study, a mutation in CC2D2A and ARL13B gene was noticed [16]. In another study, CC2D2A gene mutations were identified in the first Pakistani Family [22]. Mutation in CC2D2A accounts for 10% of the JSRD cases [22]. Bachmann-Gagescu et al. found a mutation of a similar gene in 20/209 families [23].

Patients with JS especially with breathing abnormality should receive stimulatory medications such as caffeine, supplemental oxygen, mechanical support, or tracheostomy in rare cases. Speech therapy for oromotor dysfunction, occupational and physical therapy, educational support, including special programs for the visually impaired also play an important role in patient management. In some cases, surgery may be required for polydactyly and strabismus [24]. In our case, the patient was given multivitamins, and parents were counseled and educated on other supportive therapies. No follow-up available for our patient which is a basic limitation of this case study. The authors recommend highly developed screening protocols and measures to be taken in such suspicious cases.

## Sources of funding

None.

## Ethical approval

Not required.

## Consent

Written informed consent was obtained from the patient's guardian for publication of this case report and accompanying images. A copy of the written consent is available for review by the Editor-in-Chief of this journal on request.

## Author contribution

K.S.K, I.U, and R.U.A conceived the idea; F.S, I.N, A.A, and M.S.A collected the data; M.S.A, K.S.K, R.U.A, F.S, I.U, and I.N did write up of the manuscript; and finally, I.U, M.S, M.S.A and A.A reviewed and revised the manuscript for intellectual content critically. All authors approved the final version of the manuscript.

## Registration of research studies

1. Name of the registry: Not required.
2. Unique Identifying number or registration ID: N/A
3. Hyperlink to your specific registration (must be publicly accessible and will be checked):

## Guarantor

Muhammad Sohaib Asghar.

## Provenance and peer review

Externally peer reviewed not commissioned.

## Declaration of competing interest

None.
