# Supplementary material for: Joubert syndrome a rare entity and role of radiology: A case report
Source: Ann Med Surg (Lond). 2022 Jun 30;79:104113. doi: 10.1016/j.amsu.2022.104113 (PMC9289497; doi:10.1016/j.amsu.2022.104113)
Supplement: Multimedia component 1 [file mmc1.docx]

| **SCARE 2020 Checklist** | | | |
| --- | --- | --- | --- |
| **Topic** | **Item** | **Checklist Item Description** | **Page Number** |
| **Title** | **1** | - The words ‘case report’ should appear in the title. The title should also describe the area of focus (e.g. presentation, patient population, diagnosis, surgical intervention or outcome). | 1 |
| **Key Words** | **2** | - Include three to six keywords that identify what is covered in the case report (e.g. patient population, diagnosis or surgical intervention). - Include ‘case report’ as one of the keywords. | 1 |
| **Abstract** | **3a** | Introduction and Importance   - Describe what is important, unique or educational about the case, and what does this add to the surgical literature. | 1 |
|  | **3b** | Case Presentation   - Presenting complaints, clinical and demographic details, and the patient’s main concerns. | 1 |
|  | **3c** | Clinical Findings and Investigations   - Clinical findings, investigations performed, main differentials, and subsequent diagnosis. | 1 |
|  | **3d** | Interventions and Outcome   - Describe the rationale for choosing the intervention. - Describe what was the end result. | 1 |
|  | **3e** | Relevance and Impact   - Describe the main take-away lessons or potential implications for clinical practice (minimum of three). | 1 |
| **Introduction** | **4** | Background   - Describe briefly the area of focus and the relevant background contextual knowledge.   Rationale   - Describe why the case is different to what is already known and why it is important to report? - Is the case rare or interesting for the specific healthcare setting, population or country, or is it applicable globally?   Guidelines and Literature   - Give reference to relevant surgical literature and current standards of care, including any specific guidelines. | 1 |
| **Patient Information** | **5a** | Demographic Details   - Include de-identified demographic details of the patient (e.g. age, sex, ethnicity, occupation). - Where possible, include other useful pertinent information (e.g. body mass index, hand dominance, income, level of education, marital status). | **1** |
|  | **5b** | Presentation   - Describe the patient’s presenting complaint. - Include a collateral account of the history if relevant. - Describe the patient’s mode of presentation (e.g. self-presentation, ambulance or referred by family physician or other hospital clinicians). | 1-2 |
|  | **5c** | Past Medical and Surgical History   - Include any previous interventions and relevant outcomes. | 1-2 |
|  | **5d** | Drug History and Allergies   - Specify any acute, repeat, and discontinued medications. - Include any allergies and/or adverse reactions. | 1-2 |
|  | **5e** | Family History   - Health information regarding first-degree relatives, specifying any inheritable conditions.   Social History   - Indicate smoking, alcohol, and recreational drug use. - Level of social independence, driving status, and type of accommodation.   Review of Systems   - If appropriate, report on any other information gathered outside of the focused history. | 1-2 |
| **Clinical Findings** | **6** | - Describe the general and significant clinical findings based on initial inspection and physical examination. | 1-2 |
| **Timeline** | **7** | - Summarise the sequence of events leading up to the patient’s presentation. - Delays from presentation to diagnosis and/or intervention should be reported. - Use tables or figures to illustrate the timeline of events if needed. | 1-2 |
| **Diagnostic Assessment and Interpretation** | **8a** | Diagnostic Assessment   - Bedside (e.g. urinalysis, electrocardiography, echocardiography). - Laboratory (e.g. biochemistry, haematology, immunology, microbiology, histopathology). - Imaging (e.g. ultrasound, X-ray, CT/MRI/PET). - Invasive (e.g. endoscopy, biopsy). | 1-2 |
|  | **8b** | Diagnostic Challenges   - Where applicable, describe what was challenging about the diagnoses (e.g. access, financial, cultural). | 1-2 |
|  | **8c** | Diagnostic Reasoning   - Describe the differential diagnoses, why they were considered, and why they were excluded. | 1-2 |
|  | **8d** | Prognostic Characteristics   - Include where applicable (e.g. tumour staging). | 2 |
| **Intervention** | **9a** | Pre-Operative Patient Optimisation   - Lifestyle (e.g. weight loss). - Medical (e.g. medication review, treating any relevant pre-existing medical concerns). - Procedural (e.g. nil by mouth, enema). - Other (e.g. psychological support). | N/A |
|  | **9b** | Surgical Interventions   - Describe the type(s) of intervention(s) used (e.g. pharmacological, surgical, physiotherapy, psychological, preventative). - Describe any concurrent treatments (e.g. antibiotics, analgesia, antiemetics, venous thromboembolism prophylaxis). - Medical devices should have manufacturer and model specifically mentioned. | N/A |
|  | **9c** | Specific Details regarding Interventions   - Describe the rationale behind the treatment offered, how it was performed and time to intervention. - For surgery, include details on the intervention (e.g. anaesthesia, patient position, preparation used, use of other relevant equipment, sutures, devices, surgical stage). - The degree of novelty for a surgical technique/device should be mentioned (e.g. ‘first in human’). - For pharmacological therapies, include information on the formulation, dosage, strength, route, and duration. | 2 |
|  | **9d** | Operator Details and Setting of Intervention   - Where applicable, include operator experience and position on the learning curve, prior relevant training, and specialisation (e.g. ‘junior trainee with 3 years of surgical specialty training’). - Specify the setting in which the intervention was performed (e.g. district general hospital, major trauma centre). | N/A |
|  | **9e** | Deviation from Initial Management Plan   - State if there were any changes in the planned intervention(s), and describe these alongside the rationale (e.g. delays to intervention). | N/A |
| **Follow-Up and**  **Outcomes** | **10a** | Specify Details regarding the Follow-Up   - When (e.g. how long after discharge, frequency, maximum follow-up length at time of submission). - Where (e.g. home via video consultation, primary care, secondary care). - How (e.g. telephone consultation, clinical examination, blood tests, imaging). - Any specific long-term surveillance requirements (e.g. imaging surveillance of endovascular aneurysm repair or clinical exam/ultrasound of regional lymph nodes for skin cancer). - Any specific post-operative instructions (e.g. post-operative medications, targeted physiotherapy, psychological therapy). | N/A |
|  | **10b** | Intervention Adherence and Compliance   - Where relevant, detail how well the patient adhered to and tolerated the advice provided (e.g. avoiding heavy lifting for abdominal surgery, or tolerance of chemotherapy and pharmacological agents). - Explain how adherence and tolerance were measured. | N/A |
|  | **10c** | Outcomes   - Expected versus attained clinical outcome as assessed by the clinician. Reference literature used to inform expected outcomes. - When appropriate, include patient-reported measures (e.g. questionnaires including quality-of-life scales). | N/A |
|  | **10d** | Complications and Adverse Events   - Precautionary measures taken to prevent complications (e.g. antibiotic or venous thromboembolism prophylaxis). - All complications and adverse or unanticipated events should be described in detail and ideally categorised in accordance with the Clavien-Dindo Classification (e.g. blood loss, length of operative time, wound complications, re-exploration or revision surgery). - If relevant, was the complication reported to the relevant national agency or pharmaceutical company. - Specify the duration of time between completion of the intervention and discharge, and whether this was within the expected timeframe (if not, why not). - Where applicable, the 30-day post-operative and long-term morbidity/mortality may need to be specified. - State if there were no complications or adverse outcomes. | N/A |
| **Discussion** | **11a** | Strengths   - Describe the relevant strengths of the case. - Detail any multidisciplinary or cross-speciality relevance. | 2 |
|  | **11b** | Weaknesses and Limitations   - Describe the relevant weaknesses or limitations of the case. - For novel techniques or devices, outline any contraindications and alternatives, potential risks and possible complications if applied to a larger population. | 3 |
|  | **11c** | Relevant Literature   - Include a discussion of the relevant literature and, if appropriate, similar published cases. - Describe the implications for clinical practice guidelines and any relevant hypotheses generated. | 2-3 |
|  | **11d** | - Provide a rationale for the conclusions drawn from the case. | 2 |
|  | **11e** | Take-Away Lessons   - Outline the key clinical lessons from this case report. - Discuss any differences in approach to diagnosis or patient management which the authors might adopt in future similar cases, based on their experience of the case. | 2 |
| **Patient Perspective** | **12** | - Where appropriate, the patient should be given the opportunity to share their perspective on the intervention(s) they received (e.g. sharing quotes from a consented and anonymised interview). | N/A |
| **Informed Consent** | **13** | - The authors must provide evidence of consent, where applicable, and if requested by the journal. - State the method of consent at the end of the article (e.g. verbal or written). - If not provided by the patient, explain why (e.g. death of patient and consent provided by next of kin). If the patient or family members were untraceable then document the tracing efforts undertaken. | 2 |
| **Additional Information** | **14** | - Please state any author contributions, acknowledgments, conflicts of interest, sources of funding, and where required, institutional review board or ethical committee approval. - Disclose whether the case has been presented at a conference or regional meeting. | 3 |
| **Clinical Images and Videos** | **15** | - Where relevant and available, include clinical images to help demonstrate the case pre-, peri-, and post-intervention (e.g. radiological, histopathological, patient photographs, intraoperative images). - Where relevant and available, include a link (e.g. Google Drive, YouTube) to the narrated operative video can be included to highlight specific techniques or operative findings. - Ensure all media files are appropriately captioned and indicate points of interest to allow for easy interpretation. | 3 |
| **Referencing the Checklist** | **16** | - Include reference to the SCARE 2020 publication by stating: ‘This case report has been reported in line with the SCARE Criteria [include citation]’ at the end of the introductory section. | 2 |
